# Poly[dinitrato[μ_3_-2,4,6-tris­(4-pyrid­yl)-1,3,5-triazine]cobalt(II)]

**DOI:** 10.1107/S1600536811017661

**Published:** 2011-05-14

**Authors:** Ya-Ping Wang, Ning Zhang, Xiang He, Min Shao, Ming-Xing Li

**Affiliations:** a Department of Chemistry, College of Science, Shanghai University, Shanghai 200444, People’s Republic of China; b Laboratory for Microstructures, Shanghai University, Shanghai 200444, People’s Republic of China

## Abstract

The solvothermal reaction of Co(NO_3_)_2_ and 2,4,6-tris(4-pyridyl)-1,3,5-triazine in dimethyl­formamide/ethanol mixed solvent afforded the title coordination polymer, [Co(NO_3_)_2_(C_18_H_12_N_6_)]_*n*_, in which the Co^II^ atom is seven-coordinated by pyridyl groups of three different ligands and two chelating nitrate anions. The complex displays a nano-sized porous metal–organic framework that belongs to a (10,3) topological network.

## Related literature

For metal–organic frameworks, see: Yaghi *et al.* (2003 ▶). For 2,4,6-tris(4-pyridyl)-1,3,5-triazine (tpt) coordination polymers, see: Fujita *et al.* (2005 ▶); Li *et al.* (2008 ▶). For a related nickel–tpt–nitrato coordination polymer, see: Abrahams *et al.* (1999 ▶).
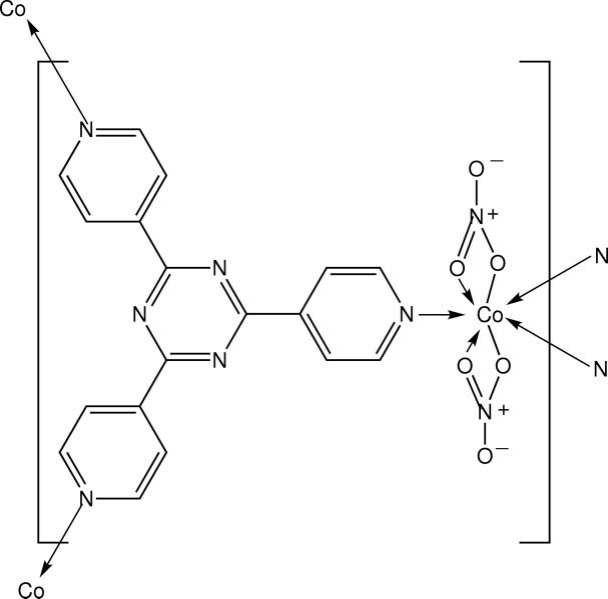


## Experimental

### 

#### Crystal data

[Co(NO_3_)_2_(C_18_H_12_N_6_)]
*M* *_r_* = 495.29
Orthorhombic, 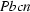

*a* = 26.193 (3) Å
*b* = 9.8005 (11) Å
*c* = 16.2950 (18) Å
*V* = 4183.0 (8) Å^3^
*Z* = 8
Mo *K*α radiation
μ = 0.88 mm^−1^
*T* = 298 K
0.20 × 0.20 × 0.10 mm

#### Data collection

Bruker SMART CCD area-detector diffractometer
Absorption correction: multi-scan (*SADABS*; Sheldrick, 2007 ▶) *T*_min_ = 0.844, *T*_max_ = 0.918
20432 measured reflections
3710 independent reflections
2756 reflections with *I* > 2σ(*I*)
*R*_int_ = 0.049

#### Refinement

*R*[*F* ^2^ > 2σ(*F* ^2^)] = 0.040
*wR*(*F* ^2^) = 0.108
*S* = 1.03
3710 reflections
298 parameters
H-atom parameters constrained
Δρ_max_ = 0.45 e Å^−3^
Δρ_min_ = −0.33 e Å^−3^

### 

Data collection: *SMART* (Bruker, 2000 ▶); cell refinement: *SAINT* (Bruker, 2000 ▶); data reduction: *SAINT*; program(s) used to solve structure: *SHELXS97* (Sheldrick, 2008 ▶); program(s) used to refine structure: *SHELXL97* (Sheldrick, 2008 ▶); molecular graphics: *SHELXTL* (Sheldrick, 2008 ▶); software used to prepare material for publication: *SHELXTL*.

## Supplementary Material

Crystal structure: contains datablocks I, global. DOI: 10.1107/S1600536811017661/tk2740sup1.cif

Structure factors: contains datablocks I. DOI: 10.1107/S1600536811017661/tk2740Isup2.hkl

Additional supplementary materials:  crystallographic information; 3D view; checkCIF report

## Figures and Tables

**Table 1:** Selected bond lengths (Å)

Co1—O1	2.231 (2)
Co1—O2	2.214 (2)
Co1—O4	2.357 (4)
Co1—O5	2.194 (3)
Co1—N3	2.128 (3)
Co1—N4^i^	2.191 (2)
Co1—N5^ii^	2.178 (2)

Symmetry codes: (i) 
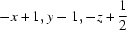
; (ii) 
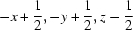
.

**Fig. 1.**
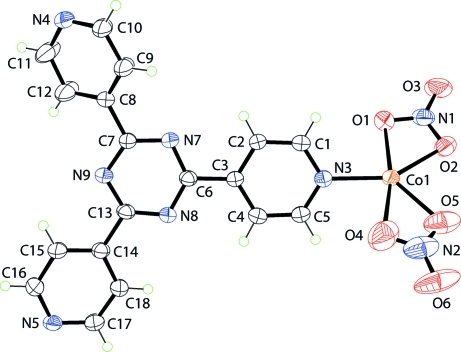
The crystallographic asymmetric unit in (I) showing atom labelling and displacement ellipsoids at the 50% probability level.

**Fig. 2.**
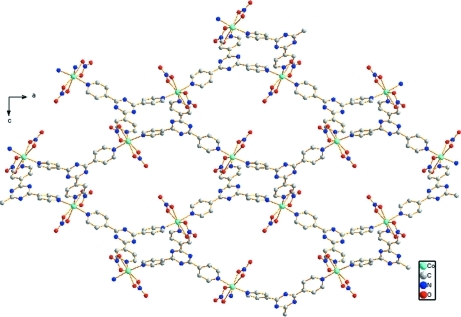
View of porous metal-organic framework in (I).

## supplementary crystallographic information

### Comment

The construction of metal-organic frameworks (MOF's) has become a very active research field in recent years, due to their intriguing structural motifs and potential applications in functional materials (Yaghi *et al.*, 2003). 2,4,6-Tris(4-pyridyl)-1,3,5-triazine (tpt) is an excellent multipyridyl ligand due to its regular trigonal structure, good rigidity and varied coordination modes. This essentially planar ligand has afforded a number of unusual and highly symmetrical coordination polymers (Fujita, *et al.*, 2005), which often display porous metal-organic frameworks enclosing nano-sized cages, cavities, chambers, and channels (Li, *et al.*, 2008). By solvothermal reaction of Co(NO_3_)_2_ with tpt, we have prepared a porous metal-organic framework [Co(tpt)(NO_3_)_2_]*_n_* (I). Herein, we report its synthesis and crystal structure.

As shown in Fig. 1, the cobalt center in (I) is seven-coordinated by three pyridyl groups from different tpt ligands and two chelating nitrates, resulting in a pentagonal-bipyramidal geometry, Table 1. Pyridyl N4^i^ and N5^ii^ occupy the axial positions defining a N4^i^-Co1-N5^ii^ bond angle of 170.62 (10) °; see Table 1 for symmetry operations. Pyridyl N3 and four nitrate oxygen donors essentially lie in an equatorial plane. One nitrate anion chelates to Co1 with similar bond lengths while the other nitrate coordinates with disparate Co—O bond distances, Table 1. Seven-coordinate Co(II) complexes are rarely observed but the long Co1-O4 distance is emphasized. Previously, a coordination polymer [Ni(tpt)(NO_3_)_2_]_n_ was reported (Abrahams, *et al.*, 1999), where Ni^II^ is six-coordinated by three oxygen atoms from monodentate and bidentate nitrates as well as three pyridyl nitrogens.

Tpt acts as an exo-tridentate ligand to connect three Co^II^ atoms through three pyridyl N-donors. This results in a 3D metal-organic framework as shown in Fig. 2. The coordination polymer shows two types of rings. One is a four-metal macrocycle containing four tpt ligands. The other one is a two-metal cycle containing two tpt ligands. From the viewpoint of network topology, the tpt ligand can be represented by a 3-connector, while Co^II^ atom is a 3-connecting node. So the polymeric network can be simplified to a (10,3) topological network. Interestingly, the packing diagram shows a nano-sized porous metal-organic framework. The approximate dimensions of the pores are 1.3 nm × 1.3 nm.

### Experimental

A mixture of Co(NO_3_)_2_.6H_2_O (29.1 mg, 0.1 mmol), tpt (31.2 mg, 0.1 mmol) and 7 ml DMF/ethanol (1:6) was sealed in a 10 ml Teflon-lined stainless steel reactor, which was heated at 433 K for 72 h. The reaction mixture was cooled to room temperature at a rate of 10 K h^-1^. Pink blocks were collected by filtration, washed with ethanol and dried in air. Yield: 33.4%. Anal. calcd for C_18_H_12_CoN_8_O_6_ (%): C, 43.65; H, 2.44; N, 22.62. Found: C, 43.24; H, 2.37; N, 22.51. IR (KBr pellet, cm^-1^): 3064w, 1618m, 1578m, 1524 s, 1471 s, 1383 s, 1301 s, 1058m, 1027m, 806 s, 654m, 514m.

### Refinement

H atoms were positioned geometrically and refined using a riding model with C—H = 0.93 Å, and with *U*_iso_(H) = 1.2*U*_eq_(C).

### Crystal data

**Table:**

[Co(NO_3_)_2_(C_18_H_12_N_6_)]	*F*(000) = 2008
*M**_r_* = 495.29	*D*_x_ = 1.573 Mg m^−^^3^
Orthorhombic, *P**b**c**n*	Mo *K*α radiation, λ = 0.71073 Å
Hall symbol: -P 2n 2ab	Cell parameters from 4158 reflections
*a* = 26.193 (3) Å	θ = 2.5–24.6°
*b* = 9.8005 (11) Å	µ = 0.88 mm^−^^1^
*c* = 16.2950 (18) Å	*T* = 298 K
*V* = 4183.0 (8) Å^3^	Block, pink
*Z* = 8	0.20 × 0.20 × 0.10 mm

### Data collection

**Table:**

Bruker SMART CCD area-detector diffractometer	3710 independent reflections
Radiation source: fine-focus sealed tube	2756 reflections with *I* > 2σ(*I*)
graphite	*R*_int_ = 0.049
φ and ω scans	θ_max_ = 25.1°, θ_min_ = 2.2°
Absorption correction: multi-scan (*SADABS*; Sheldrick, 2007)	*h* = −31→26
*T*_min_ = 0.844, *T*_max_ = 0.918	*k* = −11→10
20432 measured reflections	*l* = −19→19

### Refinement

**Table:**

Refinement on *F*^2^	Primary atom site location: structure-invariant direct methods
Least-squares matrix: full	Secondary atom site location: difference Fourier map
*R*[*F*^2^ > 2σ(*F*^2^)] = 0.040	Hydrogen site location: inferred from neighbouring sites
*wR*(*F*^2^) = 0.108	H-atom parameters constrained
*S* = 1.03	*w* = 1/[σ^2^(*F*_o_^2^) + (0.0467*P*)^2^ + 3.8374*P*] where *P* = (*F*_o_^2^ + 2*F*_c_^2^)/3
3710 reflections	(Δ/σ)_max_ = 0.001
298 parameters	Δρ_max_ = 0.45 e Å^−^^3^
0 restraints	Δρ_min_ = −0.33 e Å^−^^3^

### Special details

**Table:**

Geometry. All e.s.d.'s (except the e.s.d. in the dihedral angle between two l.s. planes) are estimated using the full covariance matrix. The cell e.s.d.'s are taken into account individually in the estimation of e.s.d.'s in distances, angles and torsion angles; correlations between e.s.d.'s in cell parameters are only used when they are defined by crystal symmetry. An approximate (isotropic) treatment of cell e.s.d.'s is used for estimating e.s.d.'s involving l.s. planes.
Refinement. Refinement of *F*^2^ against ALL reflections. The weighted *R*-factor *wR* and goodness of fit *S* are based on *F*^2^, conventional *R*-factors *R* are based on *F*, with *F* set to zero for negative *F*^2^. The threshold expression of *F*^2^ > σ(*F*^2^) is used only for calculating *R*-factors(gt) *etc*. and is not relevant to the choice of reflections for refinement. *R*-factors based on *F*^2^ are statistically about twice as large as those based on *F*, and *R*- factors based on ALL data will be even larger.

### Fractional atomic coordinates and isotropic or equivalent isotropic displacement parameters (Å^2^)

**Table:**

	*x*	*y*	*z*	*U*_iso_*/*U*_eq_	
Co1	0.380905 (14)	−0.17895 (4)	0.06119 (2)	0.03151 (15)	
C1	0.41284 (12)	0.0886 (4)	0.1400 (2)	0.0534 (10)	
H1	0.4429	0.0692	0.1121	0.064*	
C2	0.41134 (12)	0.2035 (4)	0.1879 (2)	0.0543 (10)	
H2	0.4402	0.2581	0.1932	0.065*	
C3	0.36691 (11)	0.2379 (3)	0.22819 (18)	0.0329 (7)	
C4	0.32551 (12)	0.1533 (4)	0.2173 (2)	0.0453 (9)	
H4	0.2944	0.1735	0.2421	0.054*	
C5	0.33059 (12)	0.0391 (4)	0.1696 (2)	0.0493 (9)	
H5	0.3023	−0.0172	0.1634	0.059*	
C6	0.36517 (10)	0.3595 (3)	0.28256 (18)	0.0306 (7)	
C7	0.40612 (10)	0.5367 (3)	0.34072 (18)	0.0315 (7)	
C8	0.45454 (11)	0.6099 (3)	0.35741 (19)	0.0358 (7)	
C9	0.49982 (12)	0.5414 (4)	0.3548 (3)	0.0610 (11)	
H9	0.5005	0.4493	0.3409	0.073*	
C10	0.54451 (12)	0.6091 (4)	0.3727 (3)	0.0626 (12)	
H10	0.5748	0.5598	0.3712	0.075*	
C11	0.50274 (14)	0.8051 (4)	0.3941 (3)	0.0669 (12)	
H11	0.5031	0.8974	0.4072	0.080*	
C12	0.45627 (12)	0.7447 (4)	0.3782 (3)	0.0645 (12)	
H12	0.4263	0.7954	0.3816	0.077*	
C13	0.32363 (10)	0.4965 (3)	0.36998 (18)	0.0316 (7)	
C14	0.27651 (11)	0.5375 (3)	0.41360 (19)	0.0327 (7)	
C15	0.27784 (12)	0.6350 (4)	0.4739 (2)	0.0511 (10)	
H15	0.3086	0.6758	0.4881	0.061*	
C16	0.23372 (12)	0.6722 (4)	0.5132 (2)	0.0536 (10)	
H16	0.2356	0.7391	0.5535	0.064*	
C17	0.18746 (12)	0.5219 (4)	0.4389 (2)	0.0499 (9)	
H17	0.1563	0.4813	0.4265	0.060*	
C18	0.22980 (11)	0.4795 (4)	0.3962 (2)	0.0480 (9)	
H18	0.2270	0.4125	0.3561	0.058*	
N1	0.43623 (10)	−0.1599 (3)	−0.07207 (18)	0.0479 (8)	
N2	0.32636 (13)	−0.3756 (5)	0.1387 (3)	0.0779 (12)	
N3	0.37372 (9)	0.0033 (3)	0.13120 (16)	0.0383 (6)	
N4	0.54694 (9)	0.7393 (3)	0.39189 (17)	0.0392 (6)	
N5	0.18823 (9)	0.6176 (3)	0.49676 (16)	0.0360 (6)	
N6	0.36465 (9)	0.5756 (3)	0.38169 (16)	0.0375 (6)	
N7	0.40796 (9)	0.4331 (3)	0.28751 (15)	0.0329 (6)	
N8	0.32213 (9)	0.3851 (3)	0.32351 (15)	0.0322 (6)	
O1	0.42965 (8)	−0.0587 (2)	−0.02471 (14)	0.0457 (6)	
O2	0.41055 (9)	−0.2649 (3)	−0.05510 (15)	0.0544 (6)	
O3	0.46583 (11)	−0.1558 (3)	−0.12993 (18)	0.0806 (10)	
O4	0.33747 (13)	−0.2722 (4)	0.1748 (3)	0.0995 (12)	
O5	0.34510 (11)	−0.3810 (4)	0.0677 (3)	0.0866 (10)	
O6	0.29912 (12)	−0.4647 (4)	0.1665 (2)	0.1130 (14)	

### Atomic displacement parameters (Å^2^)

**Table:**

	*U*^11^	*U*^22^	*U*^33^	*U*^12^	*U*^13^	*U*^23^
Co1	0.0236 (2)	0.0317 (2)	0.0393 (3)	0.00198 (17)	−0.00248 (17)	−0.00143 (19)
C1	0.0303 (18)	0.055 (2)	0.075 (3)	−0.0103 (16)	0.0153 (17)	−0.027 (2)
C2	0.0327 (18)	0.053 (2)	0.077 (3)	−0.0157 (16)	0.0185 (17)	−0.032 (2)
C3	0.0247 (15)	0.0353 (17)	0.0385 (17)	−0.0025 (13)	0.0011 (13)	−0.0060 (14)
C4	0.0280 (17)	0.049 (2)	0.059 (2)	−0.0034 (15)	0.0099 (15)	−0.0156 (17)
C5	0.0275 (17)	0.051 (2)	0.070 (2)	−0.0092 (15)	0.0074 (16)	−0.0219 (19)
C6	0.0236 (15)	0.0348 (17)	0.0335 (16)	0.0012 (13)	−0.0003 (12)	−0.0003 (13)
C7	0.0242 (15)	0.0329 (17)	0.0375 (17)	−0.0003 (13)	0.0002 (13)	−0.0039 (14)
C8	0.0261 (16)	0.0394 (19)	0.0418 (18)	−0.0040 (13)	0.0052 (13)	−0.0082 (15)
C9	0.0279 (18)	0.047 (2)	0.108 (3)	0.0008 (16)	−0.003 (2)	−0.032 (2)
C10	0.0259 (18)	0.054 (2)	0.108 (3)	0.0025 (16)	−0.0035 (19)	−0.037 (2)
C11	0.037 (2)	0.035 (2)	0.128 (4)	−0.0017 (16)	−0.020 (2)	−0.013 (2)
C12	0.0267 (18)	0.045 (2)	0.122 (4)	0.0059 (16)	−0.014 (2)	−0.020 (2)
C13	0.0213 (14)	0.0378 (18)	0.0357 (16)	0.0022 (13)	0.0011 (12)	−0.0008 (14)
C14	0.0262 (16)	0.0313 (17)	0.0405 (17)	0.0001 (13)	0.0032 (13)	0.0001 (14)
C15	0.0251 (17)	0.054 (2)	0.074 (3)	−0.0056 (15)	0.0085 (17)	−0.024 (2)
C16	0.0354 (19)	0.055 (2)	0.070 (3)	−0.0054 (17)	0.0116 (17)	−0.028 (2)
C17	0.0266 (17)	0.058 (2)	0.065 (2)	−0.0078 (16)	0.0038 (16)	−0.020 (2)
C18	0.0298 (17)	0.056 (2)	0.058 (2)	−0.0054 (16)	0.0062 (15)	−0.0229 (19)
N1	0.0316 (15)	0.067 (2)	0.0448 (17)	−0.0062 (14)	0.0026 (13)	−0.0058 (16)
N2	0.038 (2)	0.074 (3)	0.121 (4)	0.004 (2)	−0.014 (2)	0.045 (3)
N3	0.0272 (13)	0.0413 (16)	0.0464 (16)	−0.0018 (11)	0.0000 (12)	−0.0108 (13)
N4	0.0268 (14)	0.0416 (17)	0.0493 (16)	−0.0040 (12)	−0.0020 (12)	−0.0044 (13)
N5	0.0254 (13)	0.0362 (15)	0.0464 (16)	−0.0004 (11)	0.0068 (11)	−0.0010 (13)
N6	0.0239 (13)	0.0404 (16)	0.0482 (16)	−0.0015 (11)	0.0043 (12)	−0.0095 (13)
N7	0.0247 (13)	0.0352 (15)	0.0390 (14)	−0.0035 (11)	0.0019 (11)	−0.0045 (12)
N8	0.0251 (13)	0.0356 (15)	0.0358 (14)	−0.0025 (11)	0.0037 (11)	−0.0046 (12)
O1	0.0426 (13)	0.0465 (15)	0.0481 (14)	0.0017 (11)	0.0020 (11)	−0.0023 (12)
O2	0.0508 (15)	0.0579 (16)	0.0546 (15)	−0.0161 (13)	0.0068 (12)	−0.0125 (13)
O3	0.0626 (18)	0.113 (3)	0.0658 (18)	−0.0285 (17)	0.0321 (15)	−0.0233 (18)
O4	0.076 (2)	0.065 (2)	0.157 (4)	0.0020 (19)	−0.011 (2)	0.005 (2)
O5	0.0478 (18)	0.097 (3)	0.115 (3)	−0.0097 (17)	−0.0112 (18)	0.039 (2)
O6	0.072 (2)	0.114 (3)	0.153 (3)	−0.039 (2)	−0.014 (2)	0.087 (3)

### Geometric parameters (Å, °)

**Table:**

Co1—O1	2.231 (2)	C10—N4	1.315 (4)
Co1—O2	2.214 (2)	C10—H10	0.9300
Co1—O4	2.357 (4)	C11—N4	1.326 (4)
Co1—O5	2.194 (3)	C11—C12	1.378 (5)
Co1—N3	2.128 (3)	C11—H11	0.9300
Co1—N4^i^	2.191 (2)	C12—H12	0.9300
Co1—N5^ii^	2.178 (2)	C13—N8	1.329 (4)
C1—N3	1.330 (4)	C13—N6	1.339 (4)
C1—C2	1.370 (5)	C13—C14	1.480 (4)
C1—H1	0.9300	C14—C15	1.371 (4)
C2—C3	1.378 (4)	C14—C18	1.378 (4)
C2—H2	0.9300	C15—C16	1.371 (4)
C3—C4	1.377 (4)	C15—H15	0.9300
C3—C6	1.485 (4)	C16—N5	1.333 (4)
C4—C5	1.370 (5)	C16—H16	0.9300
C4—H4	0.9300	C17—N5	1.330 (4)
C5—N3	1.338 (4)	C17—C18	1.373 (4)
C5—H5	0.9300	C17—H17	0.9300
C6—N8	1.334 (4)	C18—H18	0.9300
C6—N7	1.335 (4)	N1—O3	1.221 (4)
C7—N6	1.331 (4)	N1—O2	1.260 (4)
C7—N7	1.336 (4)	N1—O1	1.268 (4)
C7—C8	1.482 (4)	N2—O4	1.207 (5)
C8—C9	1.364 (4)	N2—O6	1.215 (5)
C8—C12	1.365 (5)	N2—O5	1.258 (5)
C9—C10	1.377 (5)	N4—Co1^iii^	2.191 (2)
C9—H9	0.9300	N5—Co1^iv^	2.178 (2)
			
N3—Co1—N5^ii^	87.31 (10)	N4—C10—H10	118.0
N3—Co1—N4^i^	101.33 (10)	C9—C10—H10	118.0
N5^ii^—Co1—N4^i^	170.62 (10)	N4—C11—C12	123.9 (3)
N3—Co1—O5	133.94 (13)	N4—C11—H11	118.1
N5^ii^—Co1—O5	85.25 (10)	C12—C11—H11	118.1
N4^i^—Co1—O5	91.23 (10)	C8—C12—C11	119.4 (3)
N3—Co1—O2	144.01 (10)	C8—C12—H12	120.3
N5^ii^—Co1—O2	89.09 (10)	C11—C12—H12	120.3
N4^i^—Co1—O2	81.77 (10)	N8—C13—N6	125.5 (3)
O5—Co1—O2	81.26 (13)	N8—C13—C14	118.2 (2)
N3—Co1—O1	86.76 (9)	N6—C13—C14	116.3 (3)
N5^ii^—Co1—O1	91.58 (9)	C15—C14—C18	117.2 (3)
N4^i^—Co1—O1	85.33 (9)	C15—C14—C13	120.8 (3)
O5—Co1—O1	138.75 (13)	C18—C14—C13	122.0 (3)
O2—Co1—O1	57.55 (9)	C14—C15—C16	119.9 (3)
N3—Co1—O4	82.06 (12)	C14—C15—H15	120.0
N5^ii^—Co1—O4	94.84 (11)	C16—C15—H15	120.0
N4^i^—Co1—O4	90.02 (11)	N5—C16—C15	123.6 (3)
O5—Co1—O4	53.52 (14)	N5—C16—H16	118.2
O2—Co1—O4	133.93 (12)	C15—C16—H16	118.2
O1—Co1—O4	166.82 (12)	N5—C17—C18	124.0 (3)
N3—C1—C2	123.8 (3)	N5—C17—H17	118.0
N3—C1—H1	118.1	C18—C17—H17	118.0
C2—C1—H1	118.1	C17—C18—C14	119.2 (3)
C1—C2—C3	119.8 (3)	C17—C18—H18	120.4
C1—C2—H2	120.1	C14—C18—H18	120.4
C3—C2—H2	120.1	O3—N1—O2	122.3 (3)
C4—C3—C2	117.1 (3)	O3—N1—O1	122.0 (3)
C4—C3—C6	122.4 (3)	O2—N1—O1	115.6 (3)
C2—C3—C6	120.4 (3)	O4—N2—O6	124.3 (6)
C5—C4—C3	119.3 (3)	O4—N2—O5	112.9 (4)
C5—C4—H4	120.4	O6—N2—O5	122.8 (5)
C3—C4—H4	120.4	C1—N3—C5	115.8 (3)
N3—C5—C4	124.1 (3)	C1—N3—Co1	121.2 (2)
N3—C5—H5	117.9	C5—N3—Co1	123.0 (2)
C4—C5—H5	117.9	C10—N4—C11	115.9 (3)
N8—C6—N7	125.3 (3)	C10—N4—Co1^iii^	118.7 (2)
N8—C6—C3	118.4 (3)	C11—N4—Co1^iii^	124.5 (2)
N7—C6—C3	116.3 (2)	C17—N5—C16	116.0 (3)
N6—C7—N7	124.9 (3)	C17—N5—Co1^iv^	121.6 (2)
N6—C7—C8	117.9 (3)	C16—N5—Co1^iv^	122.4 (2)
N7—C7—C8	117.1 (2)	C7—N6—C13	114.7 (3)
C9—C8—C12	117.1 (3)	C6—N7—C7	114.8 (2)
C9—C8—C7	120.0 (3)	C13—N8—C6	114.5 (2)
C12—C8—C7	122.8 (3)	N1—O1—Co1	92.67 (18)
C8—C9—C10	119.7 (3)	N1—O2—Co1	93.69 (19)
C8—C9—H9	120.2	N2—O4—Co1	93.4 (3)
C10—C9—H9	120.2	N2—O5—Co1	100.0 (3)
N4—C10—C9	124.0 (3)		

Symmetry codes: (i) −*x*+1, *y*−1, −*z*+1/2; (ii) −*x*+1/2, −*y*+1/2, *z*−1/2; (iii) −*x*+1, *y*+1, −*z*+1/2; (iv) −*x*+1/2, −*y*+1/2, *z*+1/2.
